# Area light source‐triggered latent angiogenic molecular mechanisms intensify therapeutic efficacy of adult stem cells

**DOI:** 10.1002/btm2.10255

**Published:** 2021-09-21

**Authors:** Yu‐Jin Kim, Sung‐Won Kim, Gwang‐Bum Im, Yeong Hwan Kim, Gun‐Jae Jeong, Hye Ran Jeon, Dong‐Ik Kim, Haeshin Lee, Sung Young Park, Sung Min Cho, Suk Ho Bhang

**Affiliations:** ^1^ School of Chemical Engineering Sungkyunkwan University Suwon Republic of Korea; ^2^ Division of Vascular Surgery, Samsung Medical Center Sungkyunkwan University School of Medicine Seoul Republic of Korea; ^3^ Department of Health Sciences and Technology, SAIHST Sungkyunkwan University Seoul Republic of Korea; ^4^ Department of Chemistry, Center for Nature‐Inspired Technology (CNiT) Korea Advanced Institute of Science and Technology (KAIST) Daejeon Republic of Korea; ^5^ Department of Chemical and Biological Engineering Korea National University of Transportation Chungju Republic of Korea

**Keywords:** human adipose‐derived stem cells, ischemic diseases, latent reaction period, red organic light‐emitting diode, therapeutic angiogenesis

## Abstract

Light‐based therapy such as photobiomodulation (PBM) reportedly produces beneficial physiological effects in cells and tissues. However, most reports have focused on the immediate and instant effects of light. Considering the physiological effects of natural light exposure in living organisms, the latent reaction period after irradiation should be deliberated. In contrast to previous reports, we examined the latent reaction period after light exposure with optimized irradiating parameters and validated novel therapeutic molecular mechanisms for the first time. we demonstrated an organic light‐emitting diode (OLED)‐based PBM (OPBM) strategy that enhances the angiogenic efficacy of human adipose‐derived stem cells (hADSCs) via direct irradiation with red OLEDs of optimized wavelength, voltage, current, luminance, and duration, and investigated the underlying molecular mechanisms. Our results revealed that the angiogenic paracrine effect, viability, and adhesion of hADSCs were significantly intensified by our OPBM strategy. Following OPBM treatment, significant changes were observed in HIF‐1α expression, intracellular reactive oxygen species levels, activation of the receptor tyrosine kinase, and glycolytic pathways in hADSCs. In addition, transplantation of OLED‐irradiated hADSCs resulted in significantly enhanced limb salvage ratio in a mouse model of hindlimb ischemia. Our OPBM might serve as a new paradigm for stem cell culture systems to develop cell‐based therapies in the future.

## 1 INTRODUCTION

Most studies on enhancing the therapeutic effect of light have focused on immediate and drastic changes in cells. However, it is essential to remember that changes in the body induced by light require a latent reaction period. For example, when ultraviolet (UV) rays hit the skin, melanin is formed in melanocytes, located in the base layer of the skin, to afford protection to the skin. This phenomenon occurs a few days after UV light exposure.^1^, ^2^ In addition, sunburn caused by excessive light occurs 4–6 h after exposure and peaks in 24 h.^3^ Although the effects of light may not always be immediate, to the best of our knowledge, no previous study has undertaken a long‐term follow‐up of the changes occurring in cells following light induction. Therefore, in this study, we attempted to assess the long‐term effects of light on stem cells.

Several studies have reported that the therapeutic effects induced by photobiomodulation (PBM) depend on the differences in wavelength and the total amount of energy.^4^, ^5^, ^6^ In general, PBM‐induced changes in the cellular microenvironment are known to be related to redox‐sensitive transcription factors. Photons from PBM are absorbed by photoacceptors in the mitochondria and induce adenosine triphosphate (ATP) production, which induces the synthesis of reactive oxygen species (ROS) and nitric oxide (NO), resulting in the expression of redox‐sensitive transcription factors.^7^ Upregulated expression of redox‐sensitive transcription factors leads to an increase in growth factor secretion, cell proliferation, and cell migration, which supports tissue regeneration.^8^, ^9^, ^10^, ^11^, ^12^, ^13^, ^14^ However, as most PBM strategies are employed for damaged tissues composed of different cell types, identifying specific mechanisms that mediate tissue regeneration becomes challenging. Moreover, previous PBM strategies required repeated exposure to induce an appropriate therapeutic effect owing to the limited transmission of light.^8^, ^9^, ^10^, ^11^, ^12^, ^13^, ^15^ In addition, disparate results have been reported with PBM strategies employing identical energy and light sources due to the differences in power density and duration (given that energy and power density are reciprocally related).^16^, ^17^, ^18^ Therefore, it is necessary to investigate optimal PBM parameters with a clear understanding of the underlying molecular mechanisms, considering the latent reaction period to improve the therapeutic effect of organic light‐emitting diode (OLED)‐based PBM (OPBM) in the context of tissue regeneration.

**FIGURE 1:**
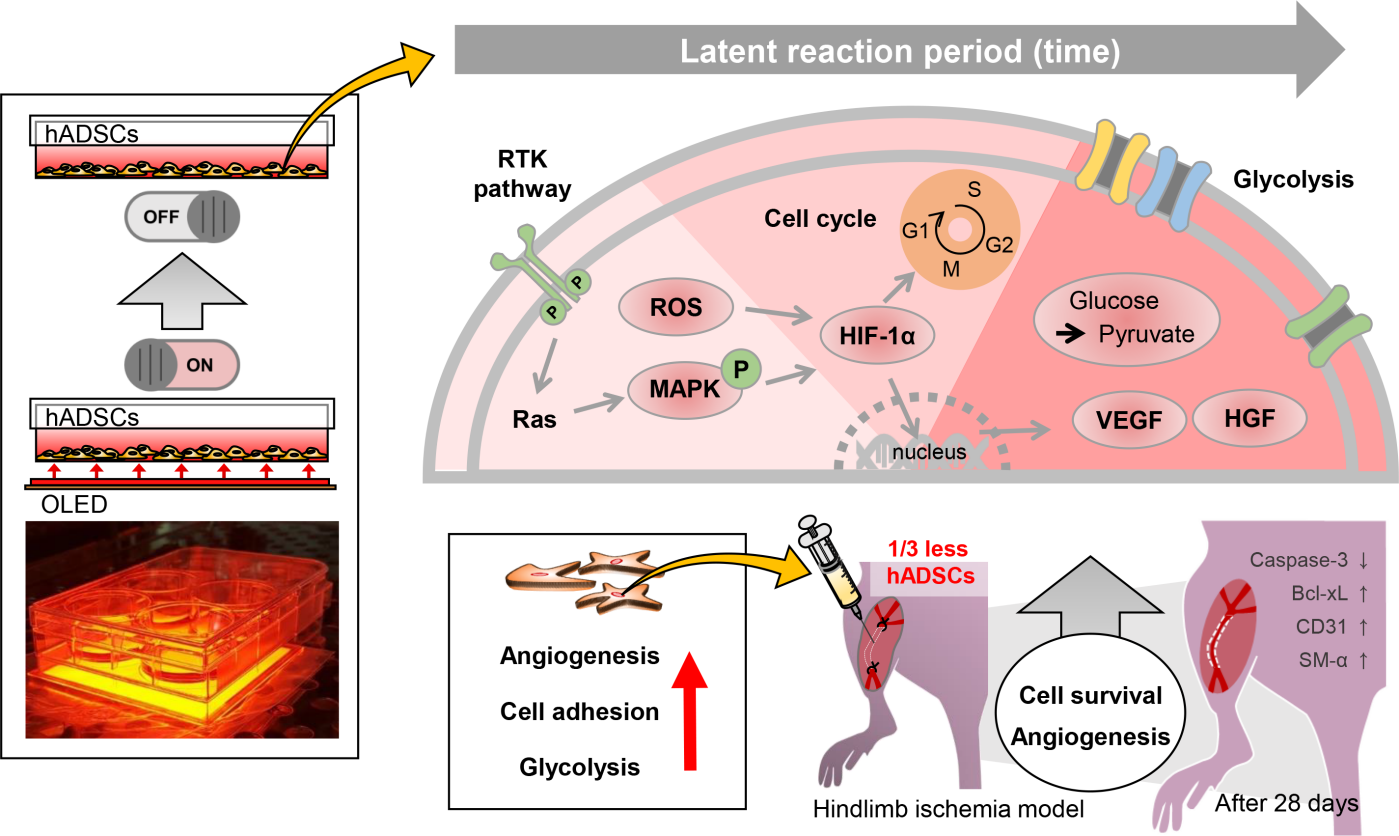
Schematic diagram depicting hADSCs subjected to red light. Schematic diagram depicting the OPBM of hADSCs. Bcl‐xL, B‐cell lymphoma‐extra‐large; CD31; cluster of differentiation 31; hADSCs, human adipose‐derived stem cells; HIF‐1α, hypoxia‐inducible factor 1‐alpha; HGF, hepatocyte growth factor; SM‐α‐Actin, smooth muscle alpha‐Actin; OLED, organic light‐emitting diode; OPBM, OLED‐based photobiomodulation; VEGF, vascular endothelial growth factor

In the present study, we established optimal irradiation parameters to subject human adipose‐derived stem cells (hADSCs) to OPBM and investigated the molecular mechanisms that can potentially enhance angiogenesis in ischemic tissues (Figure 1). We focused on improving the angiogenic efficacy of hADSCs by directly subjecting them to OPBM rather than through the PBM of tissues with complex conformations. We hypothesized that hADSCs subjected to OPBM with optimal irradiation criteria would improve angiogenesis in a mouse model of hindlimb ischemia when compared with conventional hADSCs that have not been subjected to OPBM. We optimized the parameters for the OPBM of hADSCs (wavelength, voltage, current, luminance, and duration), which had not been previously performed. Next, hADSCs were cultured and irradiated using the optimized OPBM strategy, and the molecular mechanisms related to angiogenesis were examined. Angiogenic paracrine factor expression and various OPBM‐regulated aspects, including cell viability, adhesion, apoptotic activity, cell cycle, and glycolysis, were investigated. Unlike previous PBM studies that focused on exploring the molecular mechanisms in mitochondria, we focused on receptor tyrosine kinase (RTK)‐mediated signaling mechanisms at the cell surface. To the best of our knowledge, this is the first study to evaluate the expression of glycolysis‐related enzymes induced by OPBM. Finally, hADSCs subjected to OPBM were transplanted into a mouse model of hindlimb ischemia, and angiogenesis was compared with that observed in response to conventional hADSC treatment. To confirm that OPBM enhanced the angiogenic efficacy of hADSCs, the number of hADSCs was reduced to one‐third of that used in conventional hADSC transplantation.

## 2 RESULTS

### 2.1 Enhanced secretion of angiogenic paracrine factors and adhesion ability of hADSCs without cytotoxicity following optimization of OPBM

**FIGURE 2:**
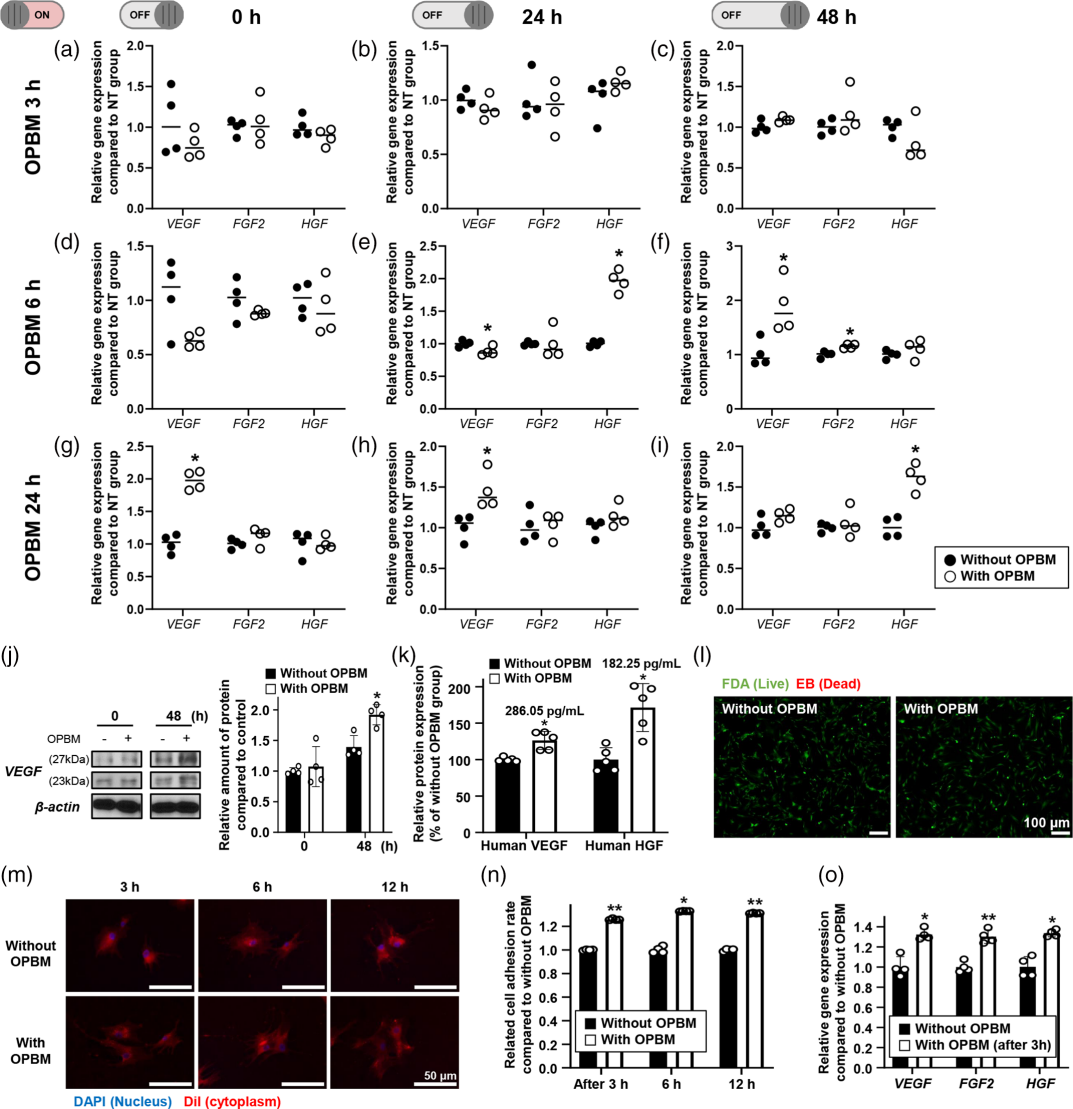
Enhanced expression of angiogenic paracrine factors and adhesion of hADSCs, without cytotoxicity following optimization of OPBM. (a–i) To determine the optimal duration of OPBM for hADSCs, the relative expression of the genes coding for angiogenic paracrine factors (*VEGF*, *FGF2*, and *HGF*) was evaluated by qRT‐PCR. Results show the gene expression patterns following the OPBM of hADSCs for 3 (a–c), 6 (d–f) or 24 h (g–i). For each OPBM duration, relative gene expression after 0, 24, and 48 h with or without OPBM, was evaluated by normalizing the values to the data collected from the without OPBM group (*p < 0.05, compared with hADSCs without OPBM treatment at each time point, *n* = 4). We chose 24 h as an optimal irradiation duration for the OPBM of hADSCs. (j) Western blot analysis of relative VEGF expression at 0 and 48 h after the OPBM of hADSCs (*p < 0.05, compared with hADSCs without OPBM, *n* = 4). (k) Secretion of VEGF and HGF by hADSCs was evaluated after 48 h of OPBM using enzyme‐linked immunosorbent assay (ELISA). Relative protein expression is shown as a percentage compared with values obtained from the without OPBM group (*p < 0.05, compared to without OPBM treatment, *n* = 5). (l) Viability of hADSCs was evaluated using the fluorescein diacetate (FDA)‐ethidium bromide (EB) assay (live cells stain green while dead cells stain red). No red cells can be observed in either group (scale bar: 100 μm). Effect of OPBM on hADSC adhesion was analyzed using cell adhesion assays performed at 3, 6, and 12 h after re‐attachment of cells. (m) Analysis of cell adhesion ability via cytoplasmic staining of cells with DiI (red). Blue color indicates DAPI staining (nucleus) (scale bar: 50 μm). (n) The adhesion ratio was evaluated by CCK‐8 assay using the without OPBM group as a control (*p < 0.05, **p < 0.01, compared with the without OPBM treatment, *n* = 4). (o) Three hours after re‐attachment of the hADSCs, the relative expression of each gene coding for angiogenic paracrine factors (*VEGF*, *FGF2*, and *HGF*) was evaluated using qRT‐PCR (*p < 0.05, **p < 0.01 compared with the without OPBM group, *n* = 4)

Herein, we attempted to determine the optimal conditions (time) for the OPBM of hADSCs required to enhance angiogenesis. Accordingly, the expression of angiogenic paracrine factors was analyzed by quantitative real‐time polymerase chain reaction (qRT‐PCR). The expression of each angiogenic factor (vascular endothelial growth factor [VEGF], fibroblast growth factor 2 [FGF2], and hepatocyte growth factor [HGF]) from an identical number of cells varied depending on the duration of OPBM (Figure 2a–i). We observed that hADSCs subjected to OPBM for 24 h showed significantly enhanced *VEGF* and *HGF* expression when compared with those exposed for 3 and 6 h (Figure 2a–i). *FGF2* expression did not show any significant difference regardless of OPBM (Figure 2a–i). After 24 h of subjecting hADSCs to OPBM, the expression of VEGF (286.05 pg/ml) and HGF (182.25 pg/ml) increased at 48 h when compared with that in hADSCs not subjected to OPBM (Figure 2j, k). This result confirmed that 24 h of OPBM is sufficient to induce paracrine angiogenic factor secretion from hADSCs. In addition, we observed that subjecting hADSCs to OPBM for 24 h did not result in any cytotoxicity, as confirmed by the live/dead assay (Figure 2l).

A previous study revealed that PBM increases cell adhesion ability following irradiation.^19^ To verify the effect of OPBM on cell adhesion, we detached cells subjected to OPBM for 24 h (as well as control cells not subjected to OPBM) from the culture plates using trypsin and then allowed them to re‐attach. We investigated cell adhesion 3, 6, and 12 h after the initiation of re‐attachment, using 1,1′‐dioctadecyl‐3,3,3′3′‐tetramethylindocarbocyanine perchlorate (DiI) staining (qualitative analysis) and cell counting kit‐8 (CCK‐8) assay (quantitative analyses). At all analyzed time points, hADSCs subjected to OPBM showed an increase in the adhesion area in re‐adhered cells when compared with hADSCs not subjected to OPBM (1.2‐fold, Figure. 2m, n). Furthermore, 3 h after re‐attachment, hADSCs subjected to OPBM showed >1.3‐fold increase in *VEGF*, *FGF2*, and *HGF* expression, compared with hADSCs not subjected to OPBM (Figure 2o). Regardless of cell detachment, hADSCs subjected to OPBM exhibited an enhanced expression of the representative angiogenic paracrine factors at the gene level.

### 2.2 Mechanism of action underlying OPBM

**FIGURE 3:**
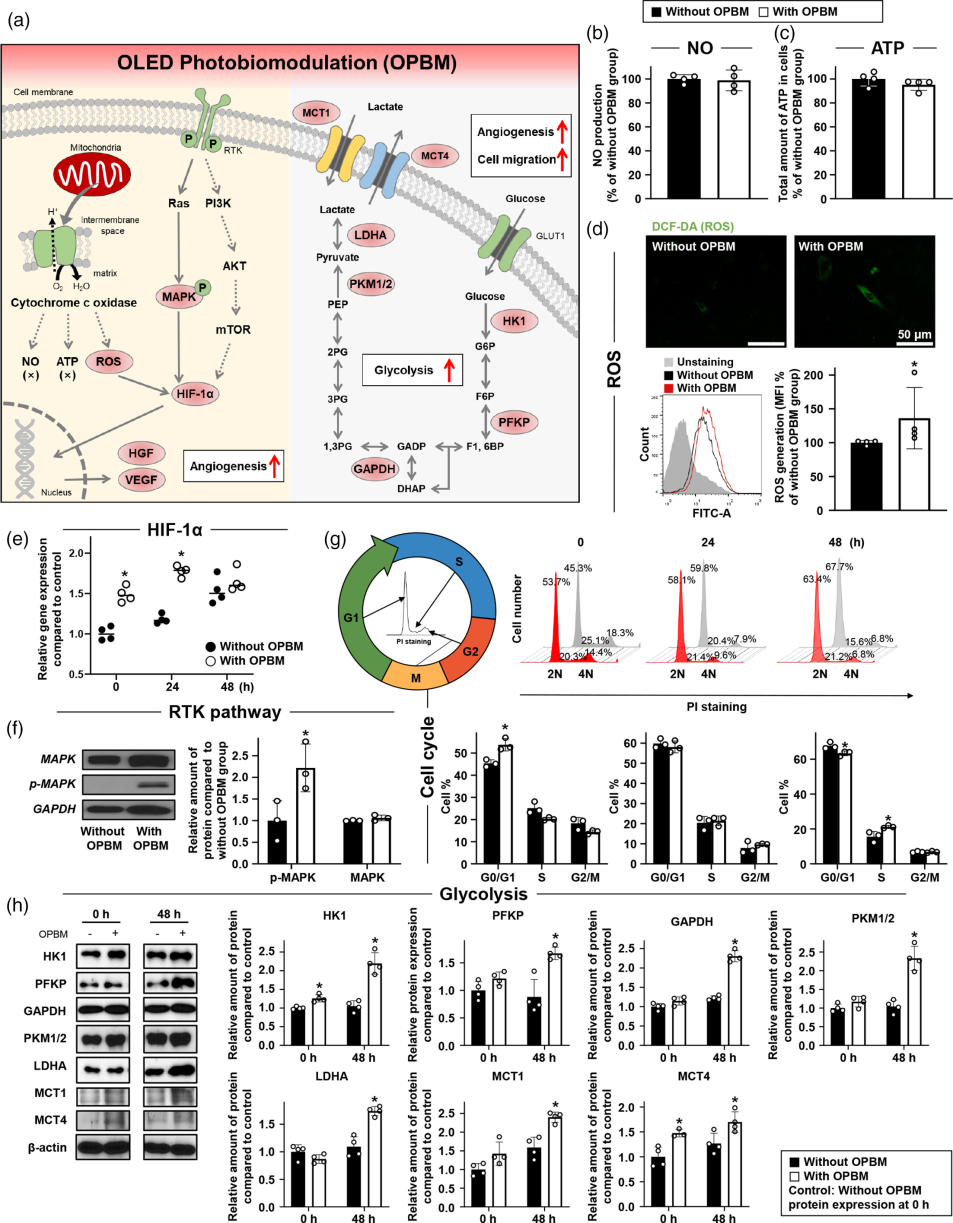
OPBM‐induced molecular mechanisms associated with angiogenesis and glycolysis enhance the therapeutic efficacy of hADSCs. (a) A schematic diagram of molecular mechanisms upregulated in hADSCs subjected to OPBM. Factors in red ovals show enhanced expression in response to OPBM. The levels of (b) NO and (c) ATP in hADSCs subjected to OPBM. The without OPBM group served as a control. (d) Staining of intracellular ROS using DCF‐DA (green), and its quantification expressed as a percent of the MFI. The without OPBM group served as a control (scale bar: 50 μm; *p < 0.05, compared with the without OPBM group, *n* = 4). (e) Relative expression of *HIF‐1α* in hADSCs with or without OPBM was analyzed using qRT‐PCR (*p < 0.05, compared with the without OPBM at each time point, *n* = 4). (f) Western blot analyses of key molecules involved in OPBM‐regulated pathways in hADSCs with or without OPBM. Quantification of the relative protein expression of the molecules (*p < 0.05, compared with the without OPBM group, *n* = 3). (g) Cell cycle distribution was evaluated using PI staining and flow cytometry (*p < 0.05, compared with the without OPBM group, *n* = 3). (h) Western blot analyses of the expression of glycolysis‐related enzymes in hADSCs with or without OPBM (*p < 0.05, compared to the without OPBM, *n* = 4)

Figure 3a is a schematic representation of the known molecular pathways and mechanisms explored in the present study. Irradiation of cells with red light at a wavelength of 610 nm stimulates the mitochondria, particularly, cytochrome c oxidase (CCO), which is the complex IV of the mitochondrial electron transport system.^7^ Upon stimulation of CCO, NO starts to dissociate from the Cu and Fe centers in CCO, and is replaced by oxygen.^20^, ^21^ This results in an increased respiration rate, which in turn, enhances ATP production^22^ and ROS levels.^14^ However, in our experiments, hADSCs subjected to OPBM exhibited different outcomes. NO production (Figure 3b) and ATP levels in hADSCs subjected to OPBM (Figure 3c) did not significantly differ from those in hADSCs not subjected to OPBM. In contrast, intracellular ROS staining revealed that ROS levels were significantly different between hADSCs subjected to OPBM and those not subjected to OPBM, as evaluated from the mean fluorescence intensity (MFI) values using 2′,7′‐dichlorodihydrofluorescein diacetate (DCF‐DA) (Figure 3d). Furthermore, we analyzed *HIF‐1α* expression by qRT‐PCR and observed that *HIF‐1α* expression was significantly increased in hADSCs up to 24 h after OPBM, compared with that in hADSCs without OPBM (Figure 3e). In addition to ROS, which can induce *HIF‐1α* expression,^23^ we also investigated the light‐induced mechanism involving RTK signaling.^23^, ^24^, ^25^, ^26^ Accordingly, we analyzed the expression of mitogen‐activated protein kinase (MAPK), phospho‐MAPK (p‐MAPK), and phosphoinositide 3‐kinase (PI3K) using western blotting. As shown in Figure 3f, MAPK expression was similar in the two treatment groups, but the expression of p‐MAPK was markedly increased (>2.5‐fold) in hADSCs subjected to OPBM when compared with that in hADSCs not subjected to OPBM. However, the expression of PI3K did not differ between hADSCs with or without OPBM (data not shown). Both factors demonstrate characteristic expression patterns when RTK on the cell surface is stimulated by light or growth factors.^23^, ^24^, ^25^, ^26^ Consistent with the findings of previous studies, our results also showed that HGF was stimulated by light (Figure 2i).^27^ Next, we investigated whether increased *HIF‐1α* expression induced changes in the cell cycle in hADSCs subjected to OPBM. HIF‐1α is known to cause cell cycle arrest at the G1 phase.^28^, ^29^, ^30^, ^31^ The ratio of cells in the G0/G1, S, and M phases was evaluated using propidium iodide (PI) staining and flow cytometric analysis (Figure 3g). Interestingly, hADSCs subjected to OPBM demonstrated an increase in G0/G1 phase cells immediately after OPBM when compared with hADSCs not subjected to OPBM. The ratio of cells in the G0/G1 phase decreased in hADSCs 48 h after OPBM treatment. However, the ratio of cells in the S phase remained unchanged in hADSCs subjected to OPBM and decreased in hADSCs not subjected to OPBM. The ratio of cells in the G2/M phase decreased over time, with no difference observed between the cells with and without OPBM at 48 h. Thus, these results suggested that OPBM affects the cell cycle by regulating *HIF‐1α* expression in hADSCs.

### 2.3 Enhanced expression of glycolytic enzymes in hADSCs subjected to OPBM

Based on the increased expression of paracrine factors associated with angiogenesis and cell cycle transition, we investigated the effects of OPBM on hADSC metabolism. To identify the changes in cellular metabolism following the OPBM of hADSCs, we analyzed the expression of enzymes involved in glycolysis as well as the levels of the metabolic product, lactate, and related factors (Figure 3h).^32^ Glycolysis is a metabolic pathway during which glucose is converted to pyruvate in the cytoplasm.^33^, ^34^, ^35^ Glucose transport across the cell membrane is facilitated by the glucose transporter 1 (GLUT1).^36^ In general, glycolysis proceeds through 10 different enzyme‐catalyzed processes. In the present study, we analyzed the following representative processes: HK1, PFKP, GAPDH, and PKM1/2 (Figure 3h). When glucose enters the cytosol, it is phosphorylated to glucose‐6‐phosphate (G6P). The enzymatic reaction associated with this step involves the phosphorylation of hexokinase1 (HK1).^37^, ^38^, ^39^ The expression of HK1 increased immediately after OPBM. Phosphofructokinase, platelet‐type (PFKP) (phosphorylates fructose‐6‐phosphate (F6P) to fructose‐1, 6‐bisphosphate (F1, 6BP)^40^), and GAPDH (reversibly phosphorylates glyceraldehyde 3‐phosphate (G3P) to 1,3‐bisphosphoglycerate (1,3‐BPG)^41^, ^42^, ^43^), did not exhibit a significant increase in expression when compared with the control (without OPBM) at 0 h; however, their expression increased 48 h after OPBM. In addition, we observed that the expression of two isozymes of pyruvate kinase (PKM1 and PKM2) was significantly enhanced 48 h after OPBM. PKM1 and PKM2 catalyze phosphate transfer from phosphoenolpyruvate (PEP) to adenosine diphosphate (ADP) to generate ATP and pyruvate.^44^, ^45^ Following glycolysis, pyruvate is reduced to lactate by lactate dehydrogenase A (LDHA).^35^, ^41^ Forty‐eight hours after OPBM, LDHA expression was significantly increased. Finally, the expression of monocarboxylate transporter 1 (MCT1) and MCT4, which are involved in the transport of lactate, increased markedly 48 h after OPBM.^46^, ^47^, ^48^, ^49^

### 2.4 Improved cell viability and angiogenic paracrine factor expression induced by OPBM after cell transplantation

**FIGURE 4:**
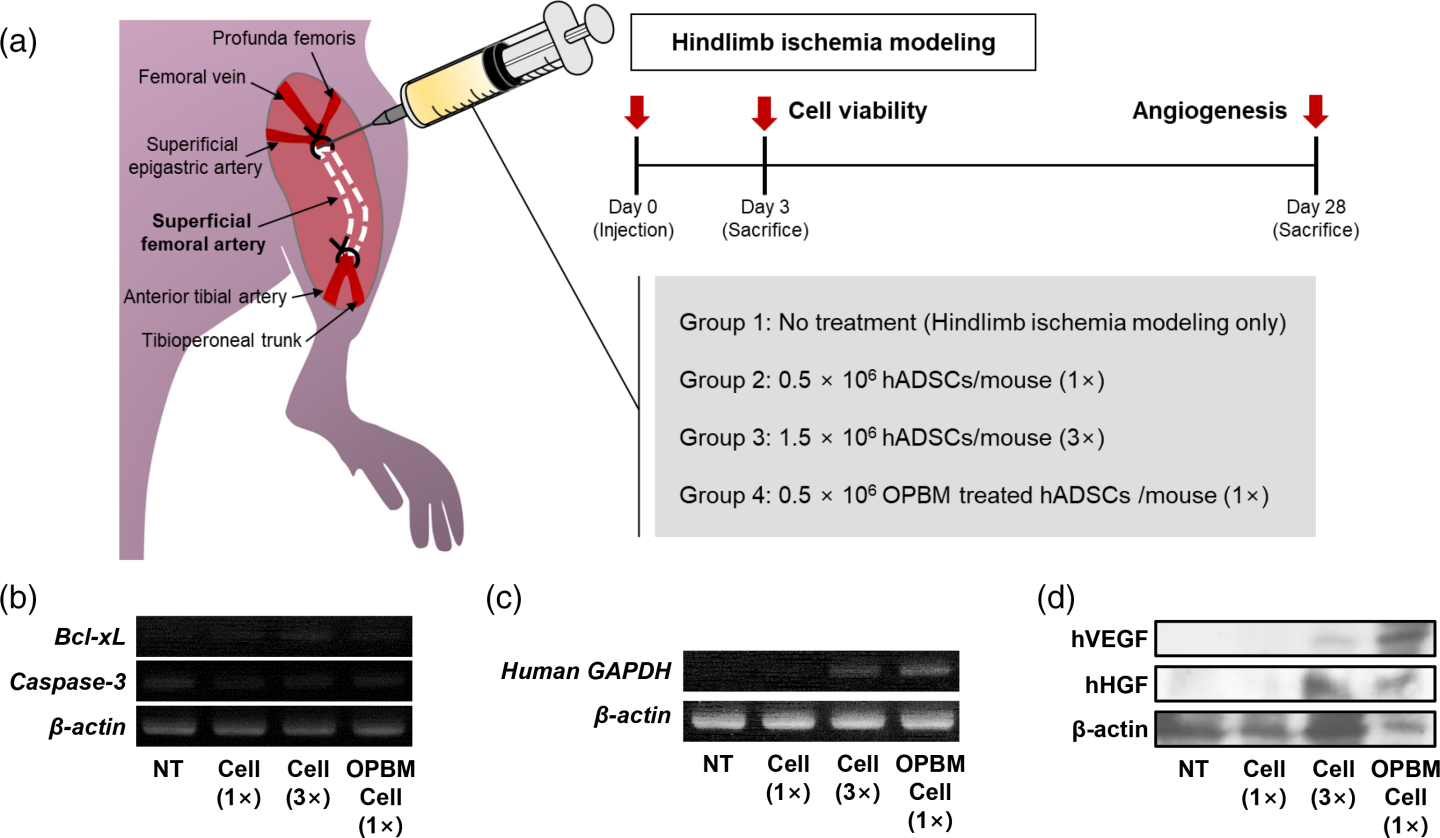
Improved cell viability of hADSCs subjected to OPBM after transplantation into ischemic tissues. (a) Schematic representation of the mouse hindlimb ischemia model. Three days after treatment, the ischemic limb tissues of mice from various groups were analyzed to determine the expression of the genes related to (b) apoptosis (mouse *Bcl‐xL* and *Caspase‐3*) and (c) human *GAPDH* by RT‐PCR. (d) Western blot analysis of the relative expression of human VEGF and HGF in the various groups following 3 days of treatment

Next, we attempted to confirm the improved angiogenic paracrine factor expression, cell adhesion, and glycolysis observed in in vitro experiments. Accordingly, we used a mouse ischemic hindlimb model. Following the induction of ischemia, the mice were treated with a reduced number of cells (0.5 × 10^6^ cells; 150 μl), the conventional number of cells (1.5 × 10^6^ cells; 150 μl), or a reduced number of cells with OPBM (0.5 × 10^6^ cells; 150 μl) by injection into the gracilis muscle (Figure 4a). The number of hADSCs injected into the ischemic tissue was selected based on the lowest or most commonly used dose for injection into ischemic hindlimbs in previous reports.^50^, ^51^, ^52^, ^53^, ^54^ Based on the results of the in vitro tests, we expected that the therapeutic effect of hADSCs subjected to OPBM would be similar or superoiro to that of hADSCs not subjected to OPBM. We divided the mice into the following four groups: no treatment (NT, ischemic hindlimb modeling only); 0.5 × 10^6^ hADSCs per mouse (cell [1×]); 1.5 × 10^6^ hADSCs per mouse (cell [3×]); 0.5 × 10^6^ OPBM‐induced hADSCs per mouse (OPBM cell [1×]). Three days after treatment, the expression of apoptosis‐related factors was analyzed using reverse transcription PCR (RT‐PCR). Compared with the NT group, the expression of B‐cell lymphoma‐extra‐large (*Bcl‐xL*; a gene related to cell survival), was increased, whereas that of *Caspase‐3* (a gene related to apoptosis) was decreased (Figure 4b) in the other three groups. Furthermore, RT‐PCR revealed that the expression of human *GAPDH*, which indicates the number of viable hADSCs transplanted into the ischemic lesions, was significantly higher in the OPBM cell (1×) group than that in the cell (3×) group (Figure 4c). In addition, hADSCs remained in the ischemic sites 3 days after the transplantation were observed through HNA staining (Figure S1). More hADSCs were observed in the cell (3×) and OPBM cell (1×) groups compared to the cell (1×) group. The expression of human VEGF and HGF in the transplanted hADSCs was analyzed using western blotting. The expression of the two representative angiogenic factors was detected in both the cell (3×) and OPBM cell (1×) groups. However, the OPBM cell (1×) group showed markedly higher levels of human VEGF and HGF expression when compared with the cell (3×) group (Figure 4d).

### 2.5 Improved ischemic limb salvage and angiogenesis in lesions following the injection of hADSCs subjected to OPBM

**FIGURE 5:**
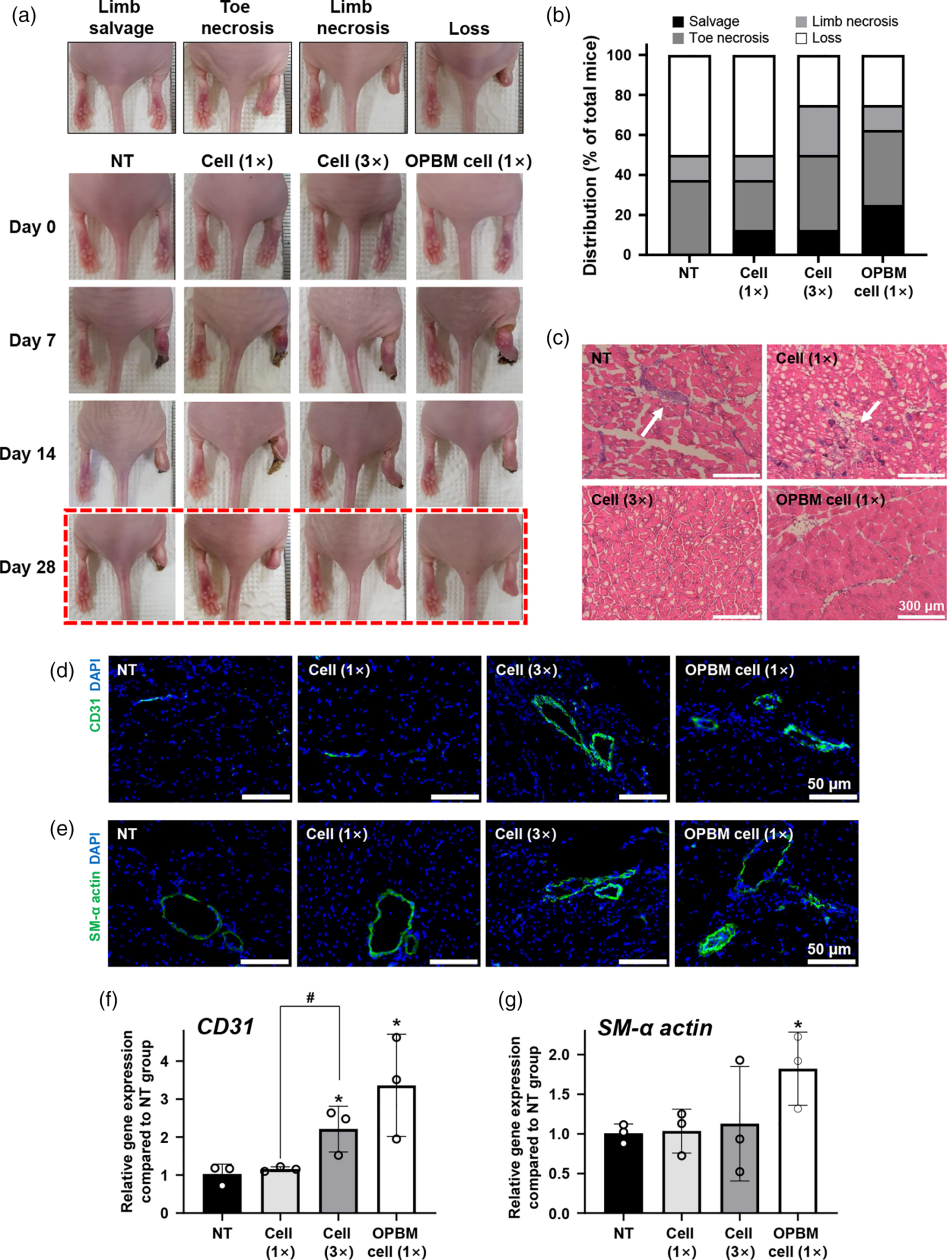
Enhanced limb salvage, angiogenesis, and decreased muscle degeneration in mice injected with hADSCs subjected to OPBM. (a) Representative photographs of limbs in each group taken 0, 7, 14, and 28 days after various treatments. (b) Physiological status of hindlimb ischemia 28 days after the various treatments. (c) Representative H&E‐stained images of the tissue sections from the hindlimb ischemic models 28 days after various treatments (scale bar: 300 μm, white arrows indicate fibrotic tissues). Immunohistochemistry for (d) CD31 (green), or (e) SM‐α‐actin (green) and DAPI (blue) staining in hindlimb ischemic region at 28 days after treatments (scale bar: 50 μm). Relative expression of (f) *CD31* and (g) *SM‐α‐Actin* in the hindlimb ischemic region 28 days after treatments. (*p < 0.05, compared with the NT, #*p* < 0.05, compared with each group, *n* = 3)

Twenty‐eight days after various treatments, the mice in each group were categorized as limb salvage, toe necrosis, limb necrosis, and limb loss based on observed limb conditions. Representative photographs of mice at 0, 7, 14, and 28 days after treatment are shown in Figure 5a. OPBM cells (1×) exhibited a significantly higher therapeutic effect when compared with cells in other groups, although the number of these cells was one‐third of that used in conventional hADSC therapy. Limb salvage was the highest in the OPBM cell (1×) group, while limb loss was similar between cell (3×) and OPBM cell (1×) groups (Figure 5b). Among the investigated groups, the conventional cell therapy (cell [1×] group) showed the lowest limb salvage and highest limb loss, similar to the NT group. Hematoxylin and eosin (H&E) staining revealed that ischemic tissues in the NT and cell (1×) groups exhibited more muscle degeneration and fibrosis than those in the cell (3×) and OPBM cell (1×) groups (Figure 5c). Angiogenesis in the ischemic tissues was markedly improved in the OPBM cell (1×) group when compared with that in the other groups (Figure 5d–g). In ischemic tissues, the number of microvessels was analyzed using immunohistochemistry (IHC) (Figure 5d and e) and qRT‐PCR (Figure 5f, g). Microvessels were most abundant in the OPBM cell (1×) group. In contrast, the lowest number of microvessels was observed in the conventional cell therapy (cell [1×]) group, similar to that in the NT group. The number of CD31^+^ microvessels was identical between the OPBM cell (1×) group and the cell (3×) group (which had three times more cells than the OPBM cell [1×] group). Interestingly, the number of SM‐α actin^+^ microvessels was higher in the OPBM cell (1×) group than in the cell (3×) group.

## 3 DISCUSSION

In the present study, we aimed to verify the intensified therapeutic efficacy of hADSCs through latent angiogenic molecular mechanisms triggered by area light sources. We demonstrated that red light at a wavelength of 610 nm from OLEDs resulted in the marked enhancement of angiogenic paracrine factor expression, cell adhesion ability, cell cycle, and glycolysis in hADSCs after exposure to optimized light irradiation. We also revealed that the viability and angiogenesis‐inducing ability of transplanted hADSCs were enhanced following OPBM treatment. Using a mouse model of hindlimb ischemia, we demonstrated that when using hADSCs subjected to OPBM for treating ischemic disease, the number of transplanted stem cells can be reduced to one‐third of the number used in the conventional method. We directly irradiated hADSCs with red light from the OLED and confirmed a significant enhancement in their angiogenic efficacy, compared with that of hADSCs without OPBM, after the latent reaction period. Importantly, due to the use of OLEDs, the properties of the light source in this study differed from those from the previous studies. Unlike lasers or LEDs, which are point light sources, OLEDs are surface light sources that can distribute light evenly over a wider area rather than focusing on a specific area. In addition, the brightness per area of OLEDs is lower than that of point light sources (laser or LED); thus, they can be used for long‐term treatments without a temperature increase that can induce cellular damage. In other words, OPBM irradiation used in our study can stimulate stem cells for an extended period and with a wider range when compared with conventional PBM, without causing cellular damage.

We optimized the duration of hADSCs exposure to OLEDs to increase angiogenic, and adhesion efficacy while decreasing cellular damage after the latent reaction period. In the present study, hADSCs showed increased expression of angiogenesis‐related genes for paracrine factors (VEGF and HGF) after 24 h of OPBM, which persisted for 48 h after irradiation. Moreover, the expression of VEGF and HGF was higher in hADSCs subjected to OPBM than in hADSCs not subjected to OPBM. In addition, we evaluated the expression of paracrine factor genes associated with angiogenesis after OPBM and trypsin‐mediated cell dissociation to confirm the persistence of OPBM effects. Interestingly, the angiogenic efficacy of hADSCs was maintained after cell dissociation, even in the absence of transfection or chemical treatment. Enhanced the expression of VEGF and HGF in hADSCs subjected to OPBM was returned to normal levels at 72 h after OPBM (Figure S2). The pattern of angiogenesis‐related gene expression in serum‐free conditions differed from that observed in serum. However, the increased expression of genes encoding angiogenic paracrine factors after OPBM was similar between the serum and serum‐free conditions (Figure S3a–c). Furthermore, the migration ability of hADSCs increased at both 3 and 24 h after OPBM in serum‐free medium (Figure S3d–g).

In contrast to previous reports focused on mitochondrial CCO,^44^, ^45^, ^55^ we observed that OPBM does not affect NO production or ATP levels. Instead, OPBM increased the intracellular level of ROS, activated the RTK receptor expressed on the cell membrane, and enhanced the therapeutic efficacy of hADSCs. As our light source, OLED, does not generate heat regardless of irradiation duration; OPBM combined with extended irradiation may have affected the RTK receptor‐mediated molecular pathways. In addition, the increased intracellular ROS level and the activated RTK pathway resulted in significantly upregulated *HIF‐1α* expression, and the subsequently angiogenic paracrine factor expression in hADSCs subjected to OPBM. Indeed, when HIF‐1α was inhibited using HIF‐1α inhibitor (CAY10585), the gene expression of *HIF‐1α*, *VEGF*, and *HGF* in the OPBM + HIF‐1α inhibitor group were markedly decreased compared to the OPBM group. Therefore, we have concluded that the upregulation of HIF‐1α expression by OPBM enhanced the intracellular ROS and MAPK activation pathways that can increase the therapeutic efficacy of hADSCs (Figure S4). Interestingly, we observed that the ratio of cells in the G0/G1, S, and G2/M phases was also altered following the OPBM of hADSCs. These results indicate that OPBM of hADSCs affects the cell cycle, which may be associated with the upregulation of *HIF‐1α*.

We analyzed the cellular metabolism in hADSCs after OPBM to elucidate the precise molecular mechanisms of action. Analysis of the expression of enzymes (HK1, PFKP, GAPDH, and PKM1/2) involved in the various steps of the glycolysis pathway 48 h after the OPBM of hADSCs revealed that levels of all enzymes were increased. Therefore, we concluded that the glycolysis pathway was activated in response to OPBM. Lactate not only affects cell proliferation, migration, and collagen synthesis, but also plays a critical role in angiogenesis and wound healing,^56^, ^57^, ^58^, ^59^, ^60^, ^61^ and is essential for the increased production of angiogenic factors such as VEGF.^62^, ^63^ Next, to investigate the levels of pyruvate, an end‐product of the glycolysis pathway, reduced to lactate by LDHA, the expression of LDHA was analyzed 48 h after OPBM. We observed that LDHA expression was increased. Furthermore, the expression of MCT1 and MCT4, which contribute to the cell‐to‐cell lactate flux,^49^, ^64^ was upregulated until 48 h after OPBM. Therefore, we concluded that lactate, produced due to the reduction of pyruvate through the activated glycolysis pathway, may induce angiogenesis and cell migration.

To confirm the therapeutic efficacy of hADSCs subjected to OPBM, we injected these cells into a mouse model of hindlimb ischemia. Three days after in vivo cell transplantation, cell survival was increased, while apoptosis was decreased in hADSCs subjected to OPBM when compared with hADSCs not subjected to OPBM. We confirmed that the OPBM cell (1×) group had a similar number of viable cells as the cell (3×) group and that each cell in the OPBM cell (1×) group expressed high levels of the angiogenic paracrine factors. Twenty‐eight days after cell transplantation, the OPBM‐induced hADSC group showed a therapeutic effect similar to that of the group injected with a 3‐fold higher number of hADSCs. Reduced fibrosis and higher expression of CD31 and SM‐α actin indicated that the OPBM of hADSCs enhanced the angiogenic efficacy of hADSCs when compared with hADSCs without OPBM. Collectively, OPBM significantly improved cell viability and angiogenesis‐inducing abilities by regulating *HIF‐1α* gene expression and glycolysis in hADSCs after the latent reaction period.

## 4 CONCLUSION

In the present study, we optimized the parameters of OPBM to enhance the expression of angiogenic paracrine factors in hADSCs and verified the underlying molecular mechanism, which differed from previous reports. Importantly, we established the criteria for PBM using a commercially available light source and optimized the customized light sources with disparate conditions. Using this OPBM strategy, we significantly enhanced the therapeutic angiogenic efficacy of hADSCs, as well as surmounted limitations associated with previous PBM studies, that is, (1) the inability to clarify the therapeutic molecular pathways, (2) the need to establish remedial protocols, (3) the thermal damage caused by heat from light sources, and (4) the challenges associated with the limited duration and area of light irradiation. Our OPBM strategy can be easily employed in conventional cell culture systems and may exhibit synergistic therapeutic effects with tissue engineering and regenerative medicine strategies based on cell therapies.

## 5 MATERIALS AND METHODS

### 5.1 Cell culture

hADSCs (Lonza, Walkersville, MD, USA) were cultured in 150‐mm culture dishes using DMEM (Gibco BRL, Gaithersburg, MD, USA) supplemented with 10% (v/v) FBS (Gibco BRL) and 1% (v/v) PS (Gibco BRL) in a 5% CO_2_ incubator at 37 °C. The culture medium was replaced every other day. For experiments, hADSCs with five to seven passages were used. The passage numbers of the hADSCs used in each experiment were identical.

### 5.2 OLED‐based PBM

The experiments were conducted using a red OLED (Kaneka, Osaka, Japan) that emitted 610 nm near‐infrared radiation. hADSCs were allowed to adhere to the culture dishes for 1 day. The next day, the nonadherent cells were washed off using phosphate‐buffered saline (PBS; Gibco BRL), and fresh culture medium was added. The cells were treated with light for 3–24 h at varying energy densities (ranging from 40 to 300 J/cm^2^).

### 5.3 qRT‐PCR

Total RNA was extracted from samples using 1 ml TRIzol (Ambion, Austin, TX, USA) and 200 μl of chloroform (Sigma, St. Louis, MO, USA). The samples were centrifuged at 12,000 rpm for 10 min at 4°C. The RNA pellet was washed with 75% (v/v) ethanol (Sigma) in water and dried. After drying, the samples were dissolved in RNase‐free water (iNtRON Biotechnology, Seoul, South Korea). Reverse transcription was performed using 1.5 μg of pure total RNA and Primescript RT Master Mix (TaKaRa, Kusatsu, Japan), followed by the PCR amplification of the synthesized cDNA. For qRT‐PCR, the SsoAdvanced Universal SYBR Green Supermix (Bio‐Rad, Hercules, CA, USA) and the CFX Connect™ real‐time PCR detection system (Bio‐Rad) were used. For the in vitro assay, qRT‐PCR was used to quantify the relative expression of *V*EGF, *FGF2*, *HGF*, and *HIF‐1α*. *GAPDH* served as an internal control. For the in vivo assays, qRT‐PCR was used to quantify the relative expression of *CD31* and *SM‐α‐*actin. *β‐*actin served as an internal control. The sequences of primers used for qRT‐PCR are listed in Table 1.

**TABLE 1:** qRT‐PCR primer sequences

Gene	Primer	Sequence (5′–3′)	NCBI sequence
*Human GAPDH* (qRT‐PCR)	Forward	GTC GGA GTC AAC GGA TTT GG	NM_001289745.3
	Reverse	GGG TGG AAT CAA TTG GAA CAT	
*Human VEGF* (qRT‐PCR)	Forward	GAG GGC AGA ATC ATC ACG AAG T	NM_001025366.3
	Reverse	CAC CAG GGT CTC GAT TGG AT	
*Human FGF2* (qRT‐PCR)	Forward	GAC GGC AGA GTT GAC GG	NM_001361665.2
	Reverse	CTC TCT CTT CTG CTT GAA GTT	
*Human HGF* (qRT‐PCR)	Forward	GAT GGC CAG CCG AGG C	NM_001010932.3
	Reverse	TCA GCC CAT GTT TTA ATT GCA	
*Human HIF‐1α* (qRT‐PCR)	Forward	CAG TTA CGT TCC TTC GAT CAG TTG	NM_181054.3
	Reverse	TTT GAG GAC TTG CGC TTT CA	
*Mouse β‐Actin* (qRT‐PCR)	Forward	GGC TGT ATT CCC CTC CAT CG	NM_007393.5
	Reverse	CCA GTT GGT AAC AAT GCC ATG T	
*Mouse Cd31* (qRT‐PCR)	Forward	CAA ACA GAA ACC CGT GGA GAT G	NM_001032378.2
	Reverse	ACC GTA ATG GCT GTT GGC TTC	
*Mouse Sm‐ α Actin* (qRT‐PCR)	Forward	CAG GCA TGG ATG GCA TCA ATC AC	NM_007392.3
	Reverse	ACT CTA GCT GTG AAG TCA GTG TCG	

Abbreviations: FGF2, fibroblast growth factor 2; HGF, hepatocyte growth factor; qRT‐PCR, quantitative real‐time polymerase chain reaction; VEGF, vascular endothelial growth factor.

### 5.4 Enzyme‐linked immunosorbent assay

Cell supernatants were collected 48 h after treatment with OPBM for 24 h. The cytokine concentration was measured using enzyme‐linked immunosorbent assay (ELISA) kits for human VEGF (R&D Systems, Minneapolis, MN, USA) and HGF (R&D Systems), according to the manufacturer's protocol.

### 5.5 Live/dead assay

A live/dead assay was performed using FDA (Sigma) and EB (Sigma). FDA (green) stains the cytoplasm of viable cells, whereas EB (red) stains the nuclei of nonviable cells. The staining solution was freshly prepared by mixing 10 ml of FDA stock solution (1.5 mg/ml of FDA in dimethyl sulfoxide), 5 ml of EB stock solution (1 mg/ml of EB in PBS), and 3 ml of PBS. The cells were then incubated with the staining solution for 3–5 min at 37°C. After staining, the samples were washed twice or thrice with PBS and examined using a fluorescence microscope (IX71, Olympus, Tokyo, Japan).

### 5.6 In vitro cell adhesion assay

In brief, cells subjected to or not subjected to OPBM (24 h) were detached using trypsin (Gibco BRL) and immediately re‐attached in 6‐ or 24‐well plates. After 3, 6, or 12 h of incubation, the unattached cells were removed by washing with PBS. After washing, the adherent cells were fixed with 4% paraformaldehyde for 10 min at room temperature, and stained with DiI (Sigma) in 6‐well plates to stain the cytoplasm of hADSCs, according to the manufacturer's instructions. The cells were counterstained with DAPI (Vector Laboratories, Burlingame, CA, USA) and examined under a fluorescence microscope (IX71, Olympus). The viability of hADSCs following OPBM was evaluated in 24‐well plates (2 × 10^4^ cells/well) using the CCK‐8 assay (Dojindo Molecular Technologies, Inc., Kumamoto, Japan; for each condition). The CCK‐8 solution was added to the cells and incubated for 2 h at 37°C. The optical density (OD) of each well was recorded at 450 nm using a microplate reader (Tecan, Mannedorf, Switzerland).

### 5.7 NO assay

The NO concentration in the culture supernatants was measured using the Griess reagent. Briefly, hADSCs were seeded in 24‐well plates (2 × 10^4^ cells/well) and incubated for 24 h. Culture supernatants were collected after 24 h of OPBM (*n* = 4). NO production was measured using the Griess Reagent System, according to the manufacturer's instructions (Promega Corp., Madison, WI, USA). The OD of each well was recorded at 560 nm using a microplate reader (Tecan).

### 5.8 ATP assay

Intracellular ATP concentrations were measured using a colorimetric ATP assay kit (Abcam, Cambridge, MA, USA, ab83355). hADSCs were seeded in 100‐mm culture dishes (10^6^ cells/dish) and incubated for 24 h. After 24 h of OPBM treatment, the cells were harvested using trypsin. ATP assay was performed according to the manufacturer's instructions.

### 5.9 ROS assay

ROS levels were measured using DCF (D339 Invitrogen, Carlsbad, CA, USA), a fluorescent indicator of ROS. In brief, hADSCs, with or without OPBM, were incubated with 10 μM DCF prepared in PBS for 20 min at 37°C. After staining, the samples were washed twice with PBS and examined under a fluorescence microscope (IX71, Olympus). The intracellular ROS concentration was also evaluated by fluorescence‐activated cell sorting (FACS) using a flow cytometer (MACSQuant® VYB, Miltenyi Biotec, Bergisch Gladbach, Germany). hADSCs, with or without OPBM, were detached using trypsin. The cells were incubated with 2.5 μM DCF prepared in PBS for 10 min at 37°C to stain intracellular ROS and then washed with FACS buffer (4% [v/v] FBS in PBS). The washed cells were resuspended in FACS buffer and analyzed by flow cytometry (MACSQuant® VYB, Miltenyi Biotec).

### 5.10 Western blotting

For the in vitro assay, 1 × 10^6^ hADSCs were subjected to OPBM for 24 h. Cells were then collected and lysed using radioimmunoprecipitation assay buffer (RIPA buffer; Rockland Immunochemicals Inc., Limerick, PA, USA). For the in vivo assay, mouse limb muscle samples were lysed in RIPA buffer using an electric homogenizer. After centrifugation at 10,000 *g* for 10 min, the supernatant was used as the protein lysate. The lysates were quantified using Quick Start Bradford 1× dye reagent (Bio‐Rad), and the absorbance was measured at 450 nm. Protein lysates were denatured by boiling in 4× Laemmli Sample buffer (Bio‐Rad) at 100°C for 10 min, separated by sodium dodecyl sulfate‐polyacrylamide gel electrophoresis (SDS‐PAGE), and transferred to nitrocellulose membranes. The membranes were blocked overnight at 4°C in TBS‐T (20 mM Tris, 137 mM NaCl, 0.1% Tween‐20; pH 7.4) containing 5% skim milk. After blocking, the membranes were incubated with the following primary antibodies: anti‐VEGFA (Abcam, ab183100), anti‐β‐actin (Sigma, A1978), anti‐MAPK (Cell Signaling, Danvers, MA, USA, 9102), anti‐p‐MAPK (Cell Signaling, 9101), anti‐GAPDH (R&D System, AF5718), anti‐HK1, anti‐PFKP, anti‐PKM1/2, anti‐LDHA (Cell Signaling, #8337), anti‐MCT1 (Santa Cruz Biotechnology, Santa Cruz, CA, USA, sc‐365501), anti‐MCT4 (Santa Cruz Biotechnology, sc‐376140), and anti‐HGF (Abcam, ab24865). The membranes were washed three times with TBS‐T for 10 min each and incubated with either of the following secondary antibodies: goat IgG HRP‐conjugated antibody (R&D System, HAF017) or rabbit IgG HRP‐conjugated antibody (R&D System, HAF008) for 1 h at room temperature. Next, the membranes were washed three times with TBS‐T for 10 min each. ProNA™ ECL Ottimo (TransLab, Daejeon, Korea) was used to visualize the protein bands. The bands were imaged and quantified using the ImageJ software (NIH).

### 5.11 Cell cycle analysis

Cell cycle distribution was analyzed using FxCycle PI/RNase staining solution (Invitrogen, F10797) and flow cytometry (MACSQuant® VYB, Miltenyi Biotec). In brief, hADSCs, subjected to or not subjected to OPBM, were trypsinized and fixed overnight in ice‐cold 70% ethanol at 4°C. The fixed cells were washed with PBS and then incubated in the FxCycle PI/RNase staining solution for 25 min at room temperature. DNA content was measured using a flow cytometer (MACSQuant® VYB, Miltenyi Biotec).

### 5.12 Mouse hindlimb ischemia model

In brief, 4‐week‐old female athymic mice (20–25 g, Orient, Seoul, South Korea) were anesthetized with xylazine (10 mg/kg) and ketamine (100 mg/kg). The femoral artery and its branches were ligated using a 6–0 silk suture (Ailee, Busan, Korea). The femoral artery was excised from the proximal branch of the external iliac artery to the distal point, where it bifurcated into the saphenous and popliteal arteries.^50^, ^51^, ^52^, ^53^, ^54^ The Institutional Animal Care and Use Committee of SKKU approved all animal treatments and experimental procedures (SKKUIACUC2019‐03‐24‐2).

### 5.13 Treatment of hind limb ischemia

Immediately after the induction of ischemia, the mice were randomly divided into four groups (*n* = 8 per group): NT, 0.5 × 10^6^ hADSCs per mouse (cell [1×]), 1.5 × 10^6^ hADSCs per mouse (cell [3×]), and 0.5 × 10^6^ OPBM‐induced hADSCs per mouse (OPBM cell (1×)]. The NT group containing mice with only arterial dissection served as the negative control group. Each group received a single injection of hADSCs into the medial thigh of the gracilis muscle on day 0. The injection doses of hADSCs were 0.5 × 10^6^ cells in 150 μl PBS per mouse (cell [1×] and OPBM cell [1×]) or 1.5 × 10^6^ cells in 150 μl PBS per mouse (cell [3×]). The hADSC doses used in this study (0.5 × 10^6^ or 1.5 × 10^6^ cells per mouse) are known to be optimal based on previous studies.^50^, ^51^, ^52^, ^53^, ^54^

### 5.14 RT‐PCR

RT‐PCR was performed using 2 × EasyTaq® PCR SuperMix (TransGen Biotech, Beijing, China) on a thermal cycler (SimpliAmp Thermal Cycler, Thermo Fisher Scientific, Waltham, MA, USA). PCR consisted of 35 cycles of denaturation at 94°C for 30 s, annealing at 55°C for 30 s, and extension at 72°C for 45 s, with a final extension at 72°C for 10 min. This was followed by electrophoresis on a 1.5% (w/v) agarose gel and visualization using RedSafe (iNtRON Biotechnology). The PCR products were analyzed using a gel documentation system (WGD‐30, Daihan Scientific, Seoul, South Korea). The RT‐PCR primer sequences are shown in Table 2.

**TABLE 2:** Real‐time polymerase chain reaction (RT‐PCR) primer sequences

Gene	Primer	Sequence (5′–3′)	NCBI sequence
*Mouse β‐Actin* (RT‐PCR)	Forward	GCT CCG GCA TGT GCA A	NM_007393.5
	Reverse	AGG ATC TTC ATG AGG TAG T	
*Mouse Bcl‐xL* (RT‐PCR)	Forward	TGG AGT AAA CTG GGG GTC GCA TCG	NM_001289717.1
	Reverse	AGC CAC CGT CAT GCC CGT CAG G	
*Mouse Caspase‐3* (RT‐PCR)	Forward	CCT CAG AGA GAC ATT CAT GG	NM_009810.3
	Reverse	GCA GTA GTC GCC TCT GAA GA	
*Human GAPDH* (RT‐PCR)	Forward	GCA CTC TTC CAG CCT TCC TTC C	NM_001101.5
	Reverse	TCA CCT TCA CCG TTC CAG TTT TT	

### 5.15 Histology and IHC

Twenty‐eight days after the treatment of mouse models with hindlimb ischemia, muscle tissue samples were obtained from the limbs and embedded in optimal cutting temperature compound (Scigen Scientific, Gardean, CA, USA). After the freezing step, the samples were cut into 10‐μm sections at −20°C. Sections containing ischemic regions were stained with H&E to assess muscle degeneration and tissue inflammation. In addition, the sections were subjected to immunofluorescence with anti‐CD31 (Abcam, ab28364) and anti‐smooth muscle α‐actin (Abcam, ab5694) antibodies. Fluorescein (FITC)‐conjugated secondary antibodies (Jackson ImmunoResearch Laboratories, West Grove, PA, USA, 111‐095‐144) were used to visualize the signals. The sections were counterstained with DAPI and examined under a fluorescence microscope (IX71, Olympus).

### 5.16 Statistical analysis

All quantitative data are expressed as mean ± SD. Statistical analysis was performed using Student's *t*‐test or one‐way ANOVA using a Bonferroni test. Statistical significance was set at *p* < 0.05.

## CONFLICT OF INTEREST

Authors declare that they have no competing interests.

## AUTHOR CONTRIBUTIONS

**Yu‐Jin Kim:** Conceptualization (equal); investigation (lead); methodology (lead); visualization (lead). **Sung‐Won Kim:** Investigation (equal). **Gwang‐Bum Im:** Investigation (equal). **Yeong Hwan Kim:** Investigation (equal). **Gun‐Jae Jeong:** Investigation (equal). **Hye Ran Jeon:** Investigation (equal). **Dong‐Ik Kim:** Conceptualization (supporting); supervision (supporting). **Sung Min Cho:** Conceptualization (equal); supervision (supporting). **Suk Ho Bhang:** Conceptualization (lead); methodology (lead); supervision (lead); visualization (supporting).

## Supporting information


**Appendix** S1: Supporting Information

## Data Availability

Data available on request from the authors
