# Supplementary material for: Area light source‐triggered latent angiogenic molecular mechanisms intensify therapeutic efficacy of adult stem cells
Source: Bioeng Transl Med. 2021 Sep 21;7(1):e10255. doi: 10.1002/btm2.10255 (PMC8780080; doi:10.1002/btm2.10255)
Supplement: Supplementary file 1 — Appendix S1: Supporting Information [file BTM2-7-e10255-s001.docx]

**Area light source-triggered latent angiogenic molecular mechanisms intensify therapeutic efficacy of adult stem cells**

*Yu-Jin Kim­^1^, Sung-Won Kim^1^, Gwang-Bum Im^1^, Yeong Hwan Kim^1^, Gun-Jae Jeong^2^, Hye Ran Jeon^3^, Dong-Ik Kim^2^, Haeshin Lee^4^, Sung Young Park^5^, Sung Min Cho^1^, Suk Ho Bhang^1,*­^*

*^1^School of Chemical Engineering, Sungkyunkwan University, Suwon 16419, Republic of Korea*

*^2^Division of Vascular Surgery, Samsung Medical Center, Sungkyunkwan University School of Medicine, Seoul 06351, Republic of Korea*

*3Department of Health Sciences and Technology, SAIHST, Sungkyunkwan University, Seoul 06351, Republic of Korea*

*4Department of Chemistry, Center for Nature-Inspired Technology (CNiT), Korea Advanced Institute of Science and Technology (KAIST), Daejeon 34141, Republic of Korea*

*5Department of Chemical and Biological Engineering, Korea National University of Transportation, Chungju 27909, Republic of Korea*

***Co-corresponding author**

Suk Ho Bhang, Ph.D.

Email: sukhobhang@skku.edu; Tel.: +82-31-290-7242; Fax: +82-31-290-7272

**Supplementary information**

**Increased expression of angiogenic paracrine factors and migration ability in hADSCs subjected to OPBM under serum-free cell culture conditions**

We investigated whether the effect of OPBM on hADSCs could be observed under serum-free conditions. Accordingly, hADSCs cultured in Dulbecco’s modified Eagle’s medium (DMEM) supplemented with 10% (v/v) fetal bovine serum (FBS) and 1% (v/v) penicillin/streptomycin (PS) were transferred to serum-free medium (DMEM supplemented with 1% (v/v) PS) 24 h prior to OPBM. The expression of the angiogenic paracrine factors (*VEGF*, *FGF2*, and *HGF*) was quantified using qRT-PCR (Fig. S3a-c). Interestingly, the expression of *FGF2*, which did not different between hADSCs with or without OPBM, was significantly different between the serum and the serum-free groups, and increased *FGF2* expression was observed until 48 h (Fig. S3b). *HGF* expression increased significantly at 24 h, but not at 48 h following the OPBM of hADSCs (Fig. S3c). Collectively, OPBM resulted in a significant upregulation of angiogenic paracrine factors in hADSCs (compared with hADSCs without OPBM), even under serum-free conditions. The scratch migration assay under serum-free conditions showed that hADSCs subjected to OPBM for 3 or 24 h showed significantly improved coverage ratios in the scraped area when compared with hADSCs without OPBM (Fig. S3d and S3e). Cell viability was evaluated using the CCK-8 assay. hADSCs subjected to OPBM for 3 h showed significantly higher viability than hADSCs without OPBM (Fig. S3f).

**Characterization of OLED and schematic of hADSC OPBM**

Instead of using previously described light sources, we used OLEDs as a new light source for OPBM to minimize light irradiation-induced cellular damage (Figure. S5). As shown in Figure Supplemental 1A (red box), a red OLED panel was installed in the culture dish. An OLED at a wavelength of 610 nm (on/off) was applied to an area of 8 × 8 cm^2^, with a thickness of 1 mm (Fig. S5b and S5c). The voltage, current, and luminance of the OLED were verified by the company. OPBM of hADSCs resulted in a significant increase in the expression of hypoxia-inducible factor 1-alpha (*HIF-1α)* through the RTK signaling pathway, and upregulation of various glycolysis pathway-related molecules. Furthermore, angiogenic paracrine factor expression, cell adhesion, and cell migration were enhanced in hADSCs subjected to OPBM compared with those not subjected to OPBM. Compared with conventional hADSC transplantation, injection of hADSCs subjected to OPBM in a mouse model of hindlimb ischemia resulted in a marked enhancement in angiogenesis and reduced apoptosis of the transplanted hADSCs.

**Materials and Methods**

*In vitro cell migration assay*

For the scratch migration assays, cells were seeded in 6-well plates and grown until they reached confluence. Prior to creating the scratch, the cells were subjected to OPBM for 3 or 24 h in serum and serum-free conditions. A linear gap was created by scratching the cell monolayer with a sterile 1000-μL tip (Neptune Scientific, San Diego, CA, USA). The wells were washed with PBS to remove detached cells, and the cells were cultured in fresh serum-free medium to prevent cell proliferation. Cell migration was observed under a microscope equipped with a 40× objective lens and expressed as the relative migration area: [(original scratched area – remaining scratched area) / original scratched area] × 100% ^1-6^.

*In vitro cytotoxicity assay*

Cell viability was evaluated using the CCK-8 assay. In brief, hADSCs were seeded in 24-well plates (2 × 10^4^ cells/well) and incubated for 24 h. The cells were subjected to OPBM at different energy densities in serum-free DMEM supplemented with 1% (v/v) PS. At 0, 24, and 48 h after OPBM in serum-free conditions, the cells were washed with PBS and incubated with the CCK-8 solution for an additional 2–3 h at 37°C. The OD of each well was recorded at 450 nm using a microplate reader (Tecan).


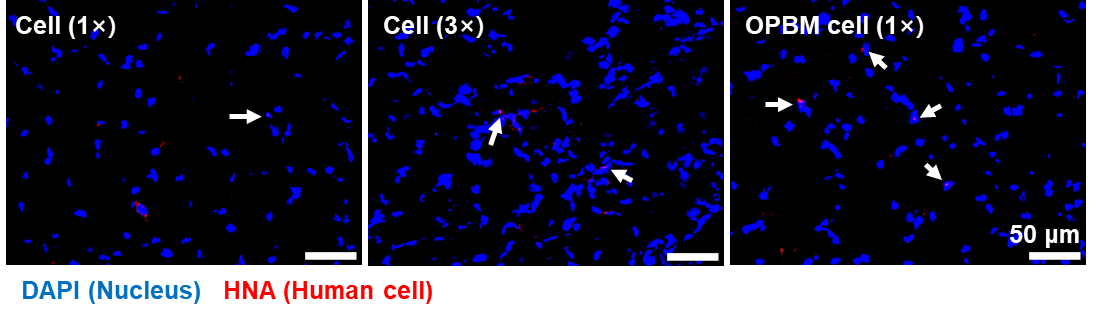


**Figure. S1.** **Enhanced cell viability in hADSCs subjected to OPBM after the transplantation into ischemic sites.** Immunohistochemistry for HNA (red, human nucleus) and DAPI (blue, nucleus) staining in hindlimb ischemic region at 3 days after the treatments (Scale bar: 50 μm).


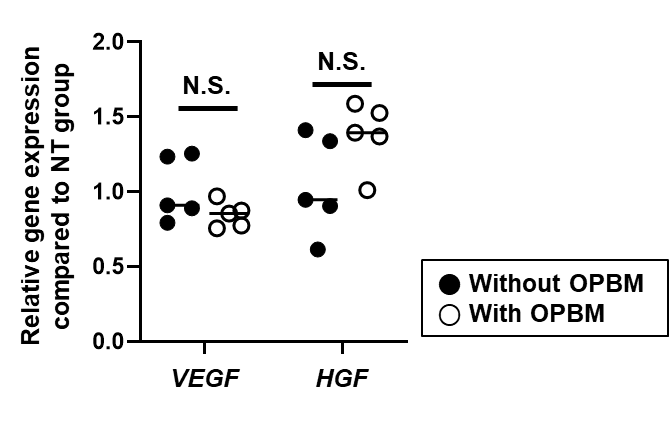


**Figure S2.** The relative expression of angiogenic paracrine factors in hADSCs after 72 h with or without OPBM (n = 5, N.S. indicates no statistical difference).


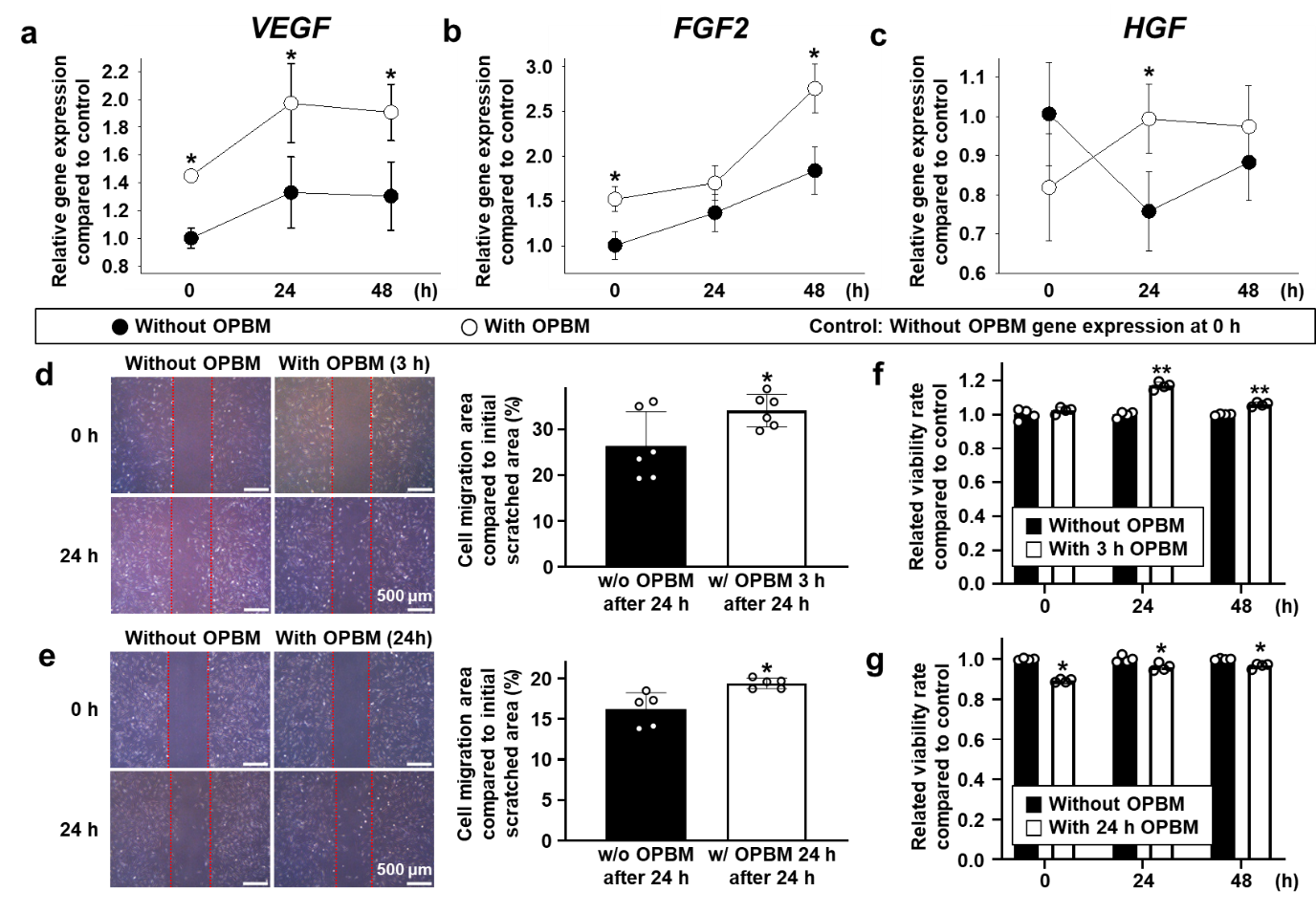


**Figure S3. Enhanced expression of angiogenic paracrine factor-coding genes and migration of hADSCs subjected to OPBM under serum-free culture conditions.** Expression of (a) *VEGF*, (b) *FGF2*, and (c) *HGF* in hADSCs subjected to OPBM and cultured in serum-free medium was analyzed by qRT-PCR (*p < 0.05, compared with the without OPBM condition at each time point, n = 4). Scratch wound coverage assay was used to analyze the migration ability of hADSCs subjected to (d) 3, or (e) 24 h of OPBM (Scale bar: 500 μm). Viability of hADSCs subjected to (f) 3, or (g) 24 h of OPBM was assessed by CCK-8 assays using the without OPBM group as a control (*p < 0.05, **p < 0.01 compared with the without OPBM group at each time point, n = 4).


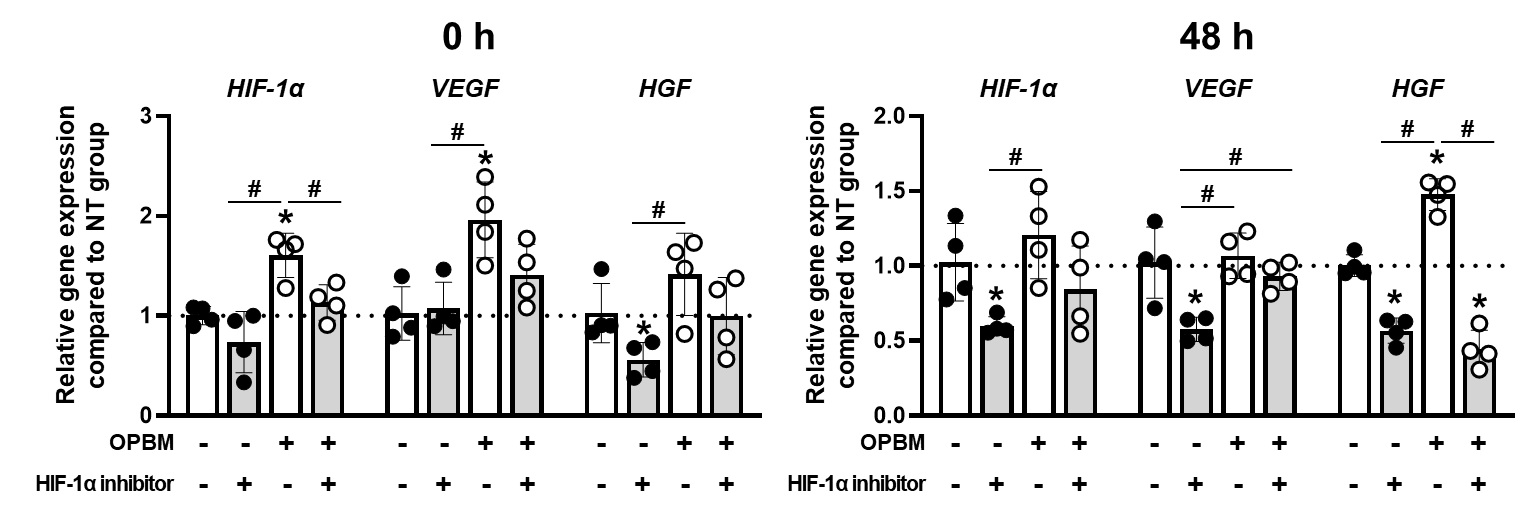


**Figure S4.** Relative expression of *HIF-1α, VEGF,* and *HGF* in hADSCs with or without OPBM and HIF-1α inhibitor (CAY10585) analyzed with qRT-PCR (*p < 0.05, compared without OPBM group, #p < 0.05, compared with each group, n = 4).


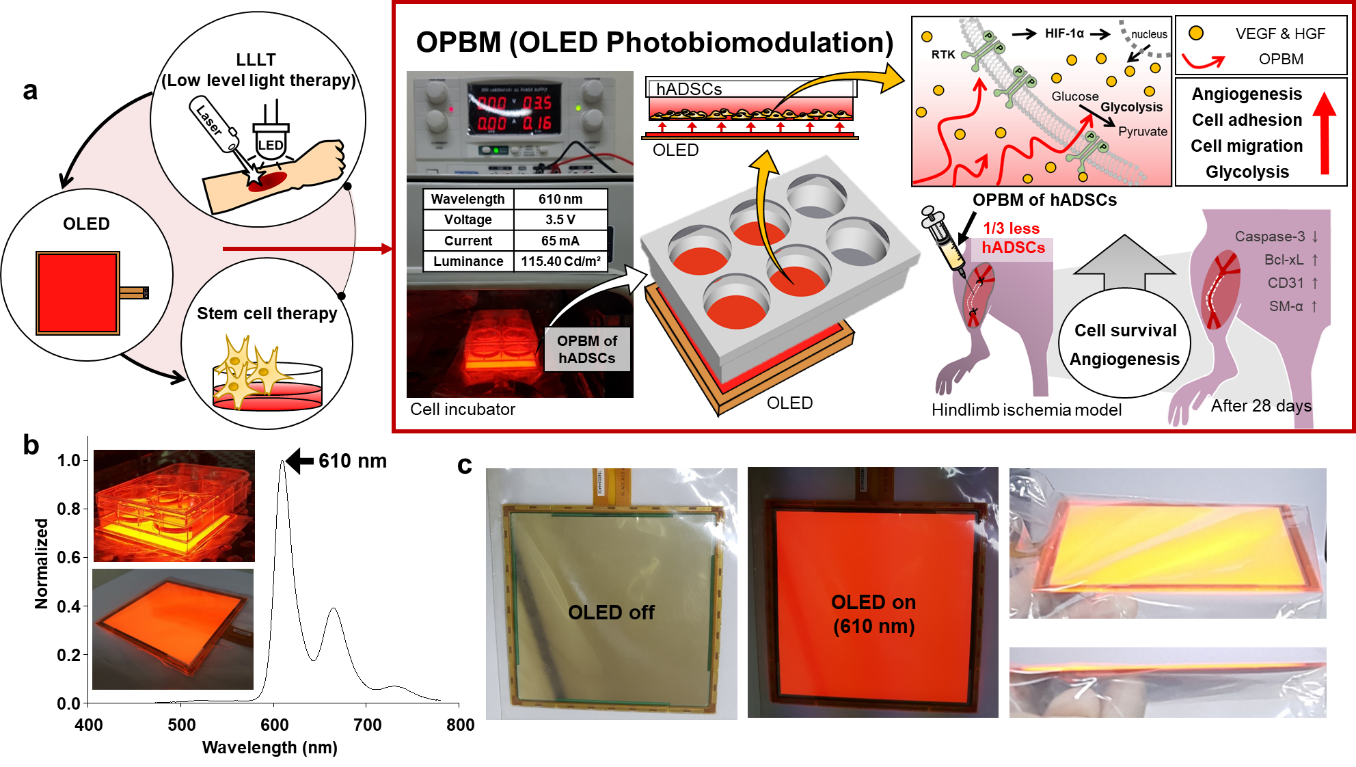


**Figure S5. Schematic diagram depicting OLED of hADSCs and the characteristics of Kaneka red OLED.** (a) Schematic diagram depicting OPBM of hADSCs. (b) Characteristics of the wavelength associated with Kaneka red OLED. (c) Photos of the front panel of the OLED in switched off (left) and on (right) modes. OLED, organic light-emitting diode; hADSCs, human adipose-derived stem cells; OPBM, OLED-based photobiomodulation; HIF-1α, hypoxia-inducible factor 1-alpha; VEGF, vascular endothelial growth factor; HGF, hepatocyte growth factor; Bcl-xL, B-cell lymphoma-extra-large; CD31, cluster of differentiation 31; SM-α-actin, smooth muscle alpha-actin.

**References**

1. Trabold O, Wagner S, Wicke C, Scheuenstuhl H, Hussain MZ, Rosen N, et al. Lactate and oxygen constitute a fundamental regulatory mechanism in wound healing. Wound Repair and Regeneration. 2003; 11: 504-9.

2. Beckert S, Farrahi F, Aslam RS, Scheuenstuhl H, Königsrainer A, Hussain MZ, et al. Lactate stimulates endothelial cell migration. Wound repair and regeneration. 2006; 14: 321-4.

3. Hunt TK, Conolly WB, Aronson SB, Goldstein P. Anaerobic metabolism and wound healing: an hypothesis for the initiation and cessation of collagen synthesis in wounds. The American Journal of Surgery. 1978; 135: 328-32.

4. Porporato PE, Payen VL, De Saedeleer CJ, Préat V, Thissen J-P, Feron O, et al. Lactate stimulates angiogenesis and accelerates the healing of superficial and ischemic wounds in mice. Angiogenesis. 2012; 15: 581-92.

5. Sun S, Li H, Chen J, Qian Q. Lactic acid: no longer an inert and end-product of glycolysis. Physiology. 2017; 32: 453-63.

6. Ghani QP, Wagner S, Becker HD, Hunt TK, Hussain MZ. Regulatory role of lactate in wound repair. Methods in enzymology: Elsevier; 2004. p. 565-75.
